# Dopamine receptor D2 regulates GLUA1-containing AMPA receptor trafficking and central sensitization through the PI3K signaling pathway in a male rat model of chronic migraine

**DOI:** 10.1186/s10194-022-01469-x

**Published:** 2022-08-10

**Authors:** Wei Zhang, Ming Lei, Qianwen Wen, Dunke Zhang, Guangcheng Qin, Jiying Zhou, Lixue Chen

**Affiliations:** 1 Laboratory Research Center, The First Affiliated Hospital of Chongqing Medical University, 1st You Yi Road, Yu Zhong District, Chongqing, 400016 China; 2 Department of Neurology, The First Affiliated Hospital of Chongqing Medical University, Chongqing, China

**Keywords:** Chronic migraine, Dopamine D2 receptor, GLUA1-containing AMPA receptor trafficking, Central sensitization

## Abstract

**Background:**

The pathogenesis of chronic migraine remains unresolved. Recent studies have affirmed the contribution of GLUA1-containing AMPA receptors to chronic migraine. The dopamine D2 receptor, a member of G protein-coupled receptor superfamily, has been proven to have an analgesic effect on pathological headaches. The present work investigated the exact role of the dopamine D2 receptor in chronic migraine and its effect on GLUA1-containing AMPA receptor trafficking.

**Methods:**

A chronic migraine model was established by repeated inflammatory soup stimulation. Mechanical, periorbital, and thermal pain thresholds were assessed by the application of von Frey filaments and radiant heat. The mRNA and protein expression levels of the dopamine D2 receptor were analyzed by qRT‒PCR and western blotting. Colocalization of the dopamine D2 receptor and the GLUA1-containing AMPAR was observed by immunofluorescence. A dopamine D2 receptor agonist (quinpirole) and antagonist (sulpiride), a PI3K inhibitor (LY294002), a PI3K pathway agonist (740YP), and a GLUA1-containing AMPAR antagonist (NASPM) were administered to confirm the effects of the dopamine D2 receptor, the PI3K pathway and GULA1 on central sensitization and the GLUA1-containing AMPAR trafficking. Transmission electron microscopy and Golgi-Cox staining were applied to assess the impact of the dopamine D2 receptor and PI3K pathway on synaptic morphology. Fluo-4-AM was used to clarify the role of the dopamine D2 receptor and PI3K signaling on neuronal calcium influx. The Src family kinase (SFK) inhibitor PP2 was used to explore the effect of Src kinase on GLUA1-containing AMPAR trafficking and the PI3K signaling pathway.

**Results:**

Inflammatory soup stimulation significantly reduced pain thresholds in rats, accompanied by an increase in PI3K-P110β subunit expression, loss of dopamine receptor D2 expression, and enhanced GLUA1-containing AMPA receptor trafficking in the trigeminal nucleus caudalis (TNC). The dopamine D2 receptor colocalized with the GLUA1-containing AMPA receptor in the TNC; quinpirole, LY294002, and NASPM alleviated pain hypersensitivity and reduced GLUA1-containing AMPA receptor trafficking in chronic migraine rats. Sulpiride aggravated pain hypersensitivity and enhanced GLUA1 trafficking in CM rats. Importantly, the anti-injury and central sensitization-mitigating effects of quinpirole were reversed by 740YP. Both quinpirole and LY294002 inhibited calcium influx to neurons and modulated the synaptic morphology in the TNC. Additional results suggested that DRD2 may regulate PI3K signaling through Src family kinases.

**Conclusion:**

Modulation of GLUA1-containing AMPA receptor trafficking and central sensitization by the dopamine D2 receptor via the PI3K signaling pathway may contribute to the pathogenesis of chronic migraine in rats, and the dopamine D2 receptor could be a valuable candidate for chronic migraine treatment.

**Supplementary Information:**

The online version contains supplementary material available at 10.1186/s10194-022-01469-x.

## Background

Chronic migraine (CM) is one of the most common headache disorders and often affects patients’ quality of life and imposes substantial individual and societal burdens [1]. The treatment of chronic migraine remains a substantial challenge due to the complex mechanisms involved.

Accumulating evidence shows that central sensitization of the trigeminal nucleus caudalis (TNC) region is a key element in the pathogenesis of chronic migraine, and persistent excitatory transmission mediated by glutamatergic neurons is generally considered to be the major factor in central sensitization [2–4]. The excitatory postsynaptic actions of glutamate, the primary excitatory neurotransmitter in the central nervous system (CNS), are carried out by its receptors, which include the N-methyl-D-aspartate receptor (NMDAR) and the α-amino-3-hydroxy-5-methyl-4-isoxazole propionic acid receptor (AMPAR) [3, 5, 6].

AMPAR consists of four subunits (GLUA1-GLUA4) [7]. Modulation of synaptic plasticity by dynamic trafficking of GLUA1-containing AMPARs has been shown to be important for neuropathic pain and noxious invasion [7, 8]. A recent study indicated that phosphorylation of the GLUA1 subunit, which is related to its recruitment to the cell membrane, may make a significant contribution to chronic migraine [9]. However, the specific molecular mechanism of GLUA1-containing AMPAR trafficking still needs to be explored.

Dopamine receptors belong to the G protein-coupled receptor (GPCR) superfamily, which is divided into two subfamilies: D1 (primarily DRD1) and D2 (primarily DRD2) [8]. Among dopamine receptors, DRD2 is one of the most important in the CNS, with roles in a series of physiological and pathological processes, such as pain, addiction, learning, and memory [10–14]. Previous research has proven that DRD2 can modulate the firing of spinal neurons and thus alleviate pain behavior in neuropathic pain models [15–17]. An in-vitro study proved that DRD2 can regulate the dendritic density and dendritic spine morphology in striatal and hippocampal neurons [18, 19]. In addition, several studies have reported that DRD2 can regulate the phosphorylation of the AMPA receptor [20] and alleviate AMPA receptor-mediated neurotoxicity [21]. These studies indicate that DRD2 might be involved in central sensitization and regulate AMPARs in chronic migraine. However, the changes in DRD2 expression and its modulatory effect on GLUA1-containing AMPARs in chronic migraine remain unclear.

The PI3K/AKT signaling pathway, a downstream pathway of GPCRs, including dopamine receptors, is actively in a wide range of physiological processes [22–24]. It has been claimed that the PI3K/AKT pathway is involved in regulating rat hippocampal long-term potentiation (LTP) as well as synaptic plasticity and increases the phosphorylation level of GLUA1-containing AMPARs [25, 26]. The PI3K pathway has also been shown to be activated in chronic migraine [26] and to have a role in the development of central sensitization in neuropathic pain [27, 28].

The PI3K pathway has been reported to be regulated by Src family kinases (SFKs), a group of tyrosine kinases participating in many cellular processes [29, 30]. Several studies have demonstrated that DRD2 can regulate the activity and level of Src family kinases in the brain [31, 32]. Moreover, we previously reported that Src family kinases could regulate the function of NMDARs and central sensitization in CM rat models [33]. In summary, we hypothesized that the dopamine D2 receptor participates in central sensitization and regulates dynamic GLUA1-containing AMPAR trafficking as well as synaptic modifications through the PI3K/AKT pathway in a Src family kinase-dependent manner in chronic migraine.

This study characterized the changes in DRD2 expression in chronic migraine and its effects on GLUA1-containing AMPAR trafficking as well as central sensitization. Specifically, we observed a significant reduction in the expression of DRD2 in the TNC and summarized that DRD2 agonist treatment can alleviate migraine-like pain behaviors and reduce central sensitization in CM rats and reduce membrane insertion of GLUA1-containing AMPARs via the PI3K/AKT pathway in a Src family kinase-dependent manner. Therefore, this study may provide a new potential therapeutic option for chronic migraine.

## Materials and methods

### Animals

Adult male Sprague‒Dawley (SD) rats (250 g-300 g; specific pathogen-free) and pregnant rats provided by Chongqing Medical University were used for this work. All procedures were carried out following the National Institutes of Health Guide for the Care and Use of Laboratory Animals. Rats were kept at room temperature (24 ± 1 °C) on a 12-h light/12-h dark cycle. No limitations for food and water accession. All rats were acclimated for 30 min before each experiment. The rats were grouped randomly except for those with an abnormal baseline pain threshold.

### Materials

All antibodies used are listed in Table 1. L-glutamine, deoxyribonuclease (DNase), poly-L-lysine (PLL), bradykinin, histamine, serotonin, and prostaglandin E2 were provided by Sigma‒Aldrich (Missouri, USA). Fluo-4 AM was purchased from Beyotime (Beijing, China). Fetal bovine serum, neurobasal medium, DMEM-HG medium, and B27 were purchased from Thermo (Waltham, USA). LY294002, sulpiride, NASPM, PP2, and 740YP were obtained from MCE (Shanghai, China), and quinpirole was purchased from Sigma‒Aldrich (Missouri, USA). Quinpirole, LY294002, PP2, sulpiride, NASPM, and 740YP were dissolved in 5% DMSO, and the inflammatory soup (IS) was produced with bradykinin (1 mM), histamine (1 mM), serotonin (1 mM), and prostaglandin E2(0.1 mM) (all from Sigma‒Aldrich, Missouri, USA), which were mixed in phosphate-buffered saline (PBS) [34, 35].

**Table 1:** Information on antibodies

Antibody	Manufacturer	Host	Dilution
Anti-ERK1/2	CST, USA	Rabbit	1:1000
Anti-p-ERK1/2	CST, USA	Rabbit	1:1000
Anti-AKT	CST, USA	Rabbit	1:1000
Anti-p-AKT	CST, USA	Rabbit	1:1000
Anti-p-Src	CST, USA	Rabbit	1:1000
Anti-p-GLUA1	Abcam, UK	Rabbit	1:1000
GAPDH	ZEN-BIOSCIENCE, China	Mouse	1:8000
Anti-GLUA1	Abcam, UK	Rabbit	1:1000
Anti-Src	CST, USA	Rabbit	1:1000
Anti-DRD2(For WB)	Proteintech, USA	Rabbit	1:1000
Anti-p-P85α	Affinity, China	Rabbit	1:1000
Anti-PI3K P110β	Bioss, China	Rabbit	1:500
Anti-PI3K P85α	Proteintech, USA	Mouse	1:10,000
Anti-PSD95	CST, USA	Mouse	1:1000
Anti-DRD2(For IF)	Santa Cruz, USA	Mouse	1:100
Anti-rabbit IgG	ZEN-BIOSCIENCE, China	Goat	1:5000
Anti-mouse IgG	ZEN-BIOSCIENCE, China	Goat	1:5000
Alexa Fluor 488 anti-mouse IgG	Beyotime, China	Goat	1: 400
Cy3 anti-rabbit IgG	Beyotime, China	Goat	1: 400

*WB* Western Blot, *IF* Immunofluorescence

### Surgery for establishing the CM model

The surgical procedure was performed as described previously [36]. First, rats under anesthetized with sevoflurane were put on a stereoscopic rack (Stoelting Co, Chicago, USA). A notch was made above the skull and the periosteum was removed to uncover the bregma. A hole with an approximate diameter of 1-mm was made over the left dura anterior fontanelle using a cranial drill (-1.0 mm posterior and + 1.5 mm lateral to the bregma). Next, a sterile catheter was placed at the eyelet with dental cement. After suturing, the rats were positioned on a warm mat until consciousness was restored. Thereafter, the rats were allowed to recover for at least 1 week to assure that their pain thresholds returned to baseline levels. During this time, the surgical area was disinfected daily with iodophor. CM rats were treated with IS (5 μL) daily for 7 days, while the IS was replaced with PBS in the Sham group.

### Animal grouping and drug delivery

As needed for experiments, rats were assigned to the following groups: Sham group, Sham + DMSO group, Sham + quinpirole group, Sham + sulpiride group, Sham + LY294002 group, Sham + PP2 group, Sham + NASPM group, CM group, CM + DMSO group, CM + quinpirole group, CM + sulpiride group, CM + LY294002 group, CM + PP2 group, CM + NASPM group, CM + quinpirole + 740YP group, CM + quinpirole + DMSO group, CM + quinpirole + sulpiride group. For the CM + quinpirole group, CM + sulpiride group, CM + PP2 group, CM + NASPM group, and CM + LY294002 group, all drugs were administered on the 7th day after IS infusion. For the CM + quinpirole + 740YP group, on Day 7 after IS injection, quinpirole was administered first, followed by 740YP 30 min later; the vehicle control was 5% DMSO. The doses of quinpirole, LY294002, PP2, sulpiride, NASPM, and 740YP were determined according to previous studies [19, 25, 33, 37, 38]. All doses of the drugs were administered by intracerebroventricular injection.

### Pain behavior test

All behavioral trials were conducted under light conditions between 09:00 and 18:00. Before the tests, the rats were acclimated to the environment for at least 30 min. Baseline testing of pain thresholds was performed before the infusion of IS or PBS. Subsequently, pain thresholds were tested daily after IS injection or after drug administration on Day 7.

First, rats were staged on a test device, and a von Frey monofilament (ranging from 1 to 26 g) was then applied vertically to the hind paw or the periorbital region (rostral area on the left or right side of the face) of each rat using an up-and-down approach to measure the mechanical threshold as described previously [34, 36].

The paw twitch latency (PWL) is thought to represent the thermal pain threshold [39]. Briefly, rats were positioned in the cage, then the radiant heat was applied from the bottom to the plantar surface of the hind paw. The time at which the rat responded to the stimulus was recorded and considered as the PWL. The maximum time was set to 25 s to protect the rats.

All tests were repeated 3 times at 5-min intervals, and the average thresholds were calculated. Throughout the experiment, the experimenter was blinded to the experimental group.

### Quantitative real-time polymerase chain reaction (qRT‒PCR)

Briefly, RNA was acquired from fresh TNC tissue using RNAiso reagent (TaKaRa, Tokyo, Japan). A PrimeScript RT Kit (TaKaRa, Tokyo, Japan) was used for reverse transcription. Then the qRT‒PCR was conducted on a thermocycler (Bio-Rad, USA) using SYBR Premix Ex Taq TM II (TaKaRa, Tokyo, Japan) to evaluate DRD1 and DRD2 mRNA expression levels. Specific primers were provided by Sangon Biotech (Shanghai, China) and are listed in Table 2. Gene expression was analyzed by the standard 2 − ΔΔCT method.

**Table 2:** Information on primers

Gene	Forward (5’—3’)	Reverse (5’–3’)
DRD1	AAGCAGCCTTCATCCTGATTAGCG	TTGTCATCCTCGGTGTCCTCCAG
DRD2	CAGTGAACAGGCGGAGAATGGATG	GTGGTGGGATGGATCAGGGAGAG
GAPDH	ATGACTCTACCCACGGCAAGCT	GGATGCAGGGATGATGTTCT

### Western blot (WB)

In brief, TNCs were removed after rats were euthanized and were then lysed with RIPA buffer containing PMSF (Beyotime, Beijing, China) to obtain total protein. A Plasma Membrane Protein Isolation and Cell Fractionation Kit (Invent Biotechnologies, USA) was applied to isolate the plasma membrane fraction. SDS-polyacrylamide gels were prepared to separate the proteins. After that, the proteins were transferred onto PVDF membranes, which were then incubated with specific primary antibodies overnight at 4 ℃ after 2 hours of blocking with 5% nonfat milk. After being incubated with secondary antibodies, the membranes were then developed by an imaging system (Fusion, Germany) using ECL reagents (Beyotime, Shanghai, China) to detect signals. Finally, the immunoblots were analyzed with ImageJ software, and the levels of the target proteins were normalized to the corresponding GAPDH level.

### Immunofluorescence staining (IF)

Rats under deep anesthesia were subjected to cardiac perfusion with PBS followed by 4% paraformaldehyde. The intact TNC was then immediately isolated and postfixed with 4% paraformaldehyde at 4 °C. After dehydration using graded concentrations of sucrose solution (20% and 30%), the tissues were sectioned using a cryostat (Leica, Japan) into 15-μm slices, which were placed on carrier slides. Antigen repair with sodium citrate (Beyotime, Beijing, China) and blocking with 10% goat serum (Boster, Beijing, China) were then executed. For fluorescence colabeling experiments, the primary antibodies were mixed and diluted with 1% PBS and were subsequently incubated with the sections at 4 °C overnight. After incubation with the fluorescent secondary antibody and counterstaining of the nuclei with 4’,6-diamidino-2-phenylindole (DAPI), a confocal laser scanning fluorescence microscope (Zeiss, Germany) was used to acquire images, which were analyzed with ImageJ.

### Golgi-Cox staining

An FD Rapid Golgi Staining Kit TM (NeuroTechnologies, USA) was utilized to observe the dendritic spines in the TNC. After rats were sacrificed, TNCs were harvested and then washed quickly with double-distilled water and immersed in a premixed solution (liquid A and liquid B at a ratio of 1:1) which was refreshed once in 24 h and then kept for 2 weeks (in a dark environment). Then the tissues were transferred to liquid C and incubated for 3 days (in a dark environment). A vibratome (Leica VT 1200S, Japan) was used to generate 150-μm-thick tissue slices, which were then stained in a mixture of liquid D, liquid E, and double-distilled water at a ratio of 1:1:2 for 10 min. Next, after being dehydrated with increasing concentrations of ethanol (50%, 75%, 95%, and 100%) and permeating with xylene, the slices were sealed with neutral resin. Images were acquired using a microscope (Axio Imager A2) and were analyzed with ImageJ. All steps were carried out at room temperature.

### Transmission Electron Microscopy (TEM)

The relevant steps can be found in our previous work [40]. Briefly, rats were anesthetized and then subjected to cardiac perfusion with PBS followed by 2.5% glutaraldehyde. TNCs were separated and cut into 1 m^3^ pieces using a blade. Next, the pieces were soaked in 4% glutaraldehyde at 4 °C and then post-treated at Chongqing Medical University. Finally, images were acquired using a JEM-1400 PLUS transmission electron microscope at 50,000 X or 30,000 X magnification and then statistically analyzed with Image-Pro 6.0.

### Culture of TNC primary neurons

TNCs were removed from embryos on Day 18 of pregnancy sterilely, and then the meninges and blood were removed as described previously [41]. Next, the tissues were digested with papain (2 mg/ml) for 25 min. The tissues were gently and repeatedly disrupted by pipetting until the large cell clumps disappeared at the end of the digestion step. After centrifugation, the cells were resuspended in DMEM-HG medium. Finally, the cells were plated (2.5 X 10^5^ cells/dish) on confocal dishes that were precoated with poly-L-lysine (PLL) at 37 °C and 5% CO2. About 4–6 h later, the DMEM-HG was replaced with neurobasal containing B27(2%) and L-glutamine (0.5 mM/L), followed by half volume changes every 3 days. Cells were incubated until Day 7 for subsequent experiments.

### Determination of intracellular Ca^2+^ concentrations

The primary neurons were pretreated with quinpirole (1 μM) [19] for 12 h and LY294002 (20 μM) [42] for 1 h at 37 °C and 5% CO2, and were then incubated with 4 μM Fluo-4-AM (150 μl/well, diluted in PBS buffer) for 30 min at 37 °C. Then, 900 μl of HBSS was left in each well after washing three times with warm (37 °C) HBSS solution. Then, 100 μl of 10X NMDA was added at a specific time (the final concentration is 50 μM), and the changes in the intracellular calcium concentration were determined using the confocal microscope described above (Zeiss, Germany) at a detection wavelength of 488 nm over a total of 500 s for each group. The fluorescence intensity in the collected images was analyzed by ZEN software [32].

### Statistical analysis

All data were analyzed, and graphs were generated using GraphPad Prism 8 (GraphPad Software Inc., San Diego, CA, USA). Data in this paper are expressed as mean ± SEM. The Kolmogorov‒Smirnov test was used to check for normality. Significant differences for two-group and multiple-group comparisons were analyzed by the independent‒sample t-test and one-way ANOVA followed by Dunnett’s multiple comparison test, respectively. Two-factor analysis followed by the Bonferroni post hoc test was used to analyze the behavioral data. *P* values < 0.05 were thought to be statistically significant.

## Results

### DRD2 expression was decreased in CM rats

**Fig. 1:**
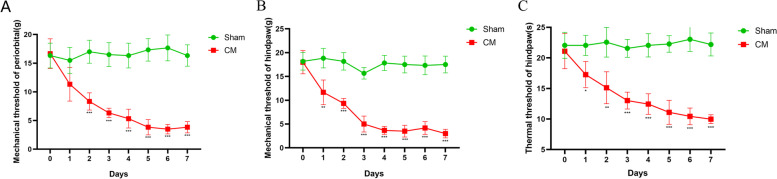
Lower pain thresholds in the CM group. **A** and **B** The hind paw mechanical and thermal pain thresholds in rats treated with IS injection were markedly lower than those in rats treated with PBS. **C** The periorbital pain threshold was markedly decreased in the CM group compared to the Sham group. Two-way ANOVA with Bonferroni post hoc test; *n* = 6/group; *P < 0.05, **P < 0 .01, ***P < 0.001 vs. the Sham group

**Fig. 2:**
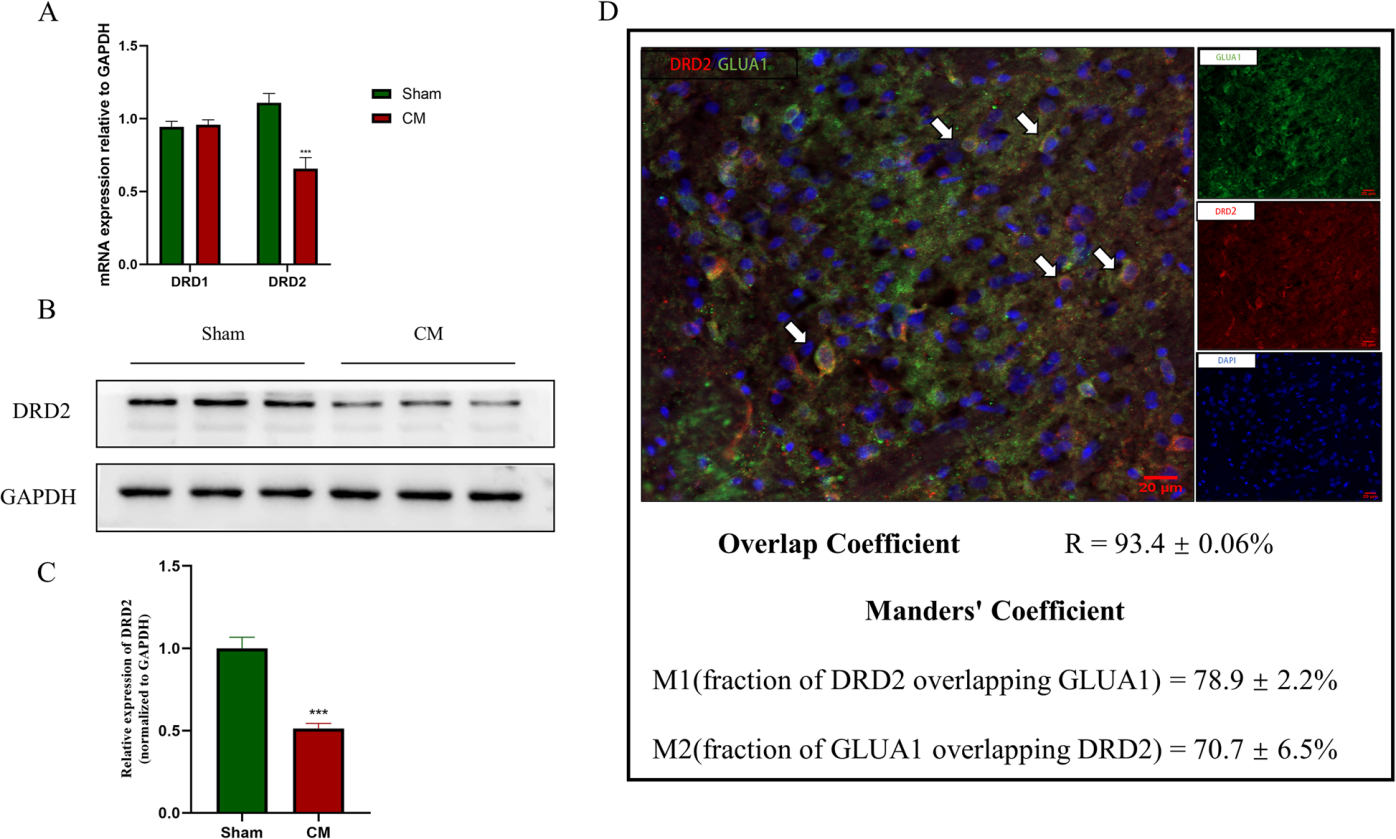
The expression of the dopamine D1 and D2 receptors in CM rats. A, The DRD2 mRNA level was markedly lower in the CM group than in the Sham group, while significant differences in DRD1 mRNA expression were not observed. **B** and **C**, The DRD2 protein level in the CM group was statistically decreased compared to that in the Sham group. Two-tailed Student’s t test; *n* = 6/group; ***P < 0.001 vs. the Sham group. **D** DRD2 (red; 78.9 ± 2.2%) and GLUA1-containing AMPARs (green; 70.7 ± 6.5%) were colocalized in the TNC in CM rats (scale bar = 20 μm; *n* = 6/group)

To determine the alterations in DRD2 in chronic migraine rats, the rat model of chronic migraine was established, and the periorbital, hind paw mechanical, and thermal pain thresholds of the model rats were evaluated, as shown in Fig. 1A-C. The observation that CM rats had lower pain thresholds than Sham rats revealed that the CM model had been successfully established. We then used qRT‒PCR to examine the expression of the dopamine receptors D1 and D2 in the TNC in CM rats, and the results showed that there was no significant change in dopamine D1 receptor mRNA expression, whereas D2 expression was downregulated in CM rats relative to Sham rats (Fig. 2A). Western Blotting confirmed that the protein level of DRD2 was downregulated in the TNC of CM rats (Fig. 2B, C).

### DRD2 agonist treatment reduces central sensitization and membrane recruitment of GLUA1 in CM rats

**Fig. 3:**
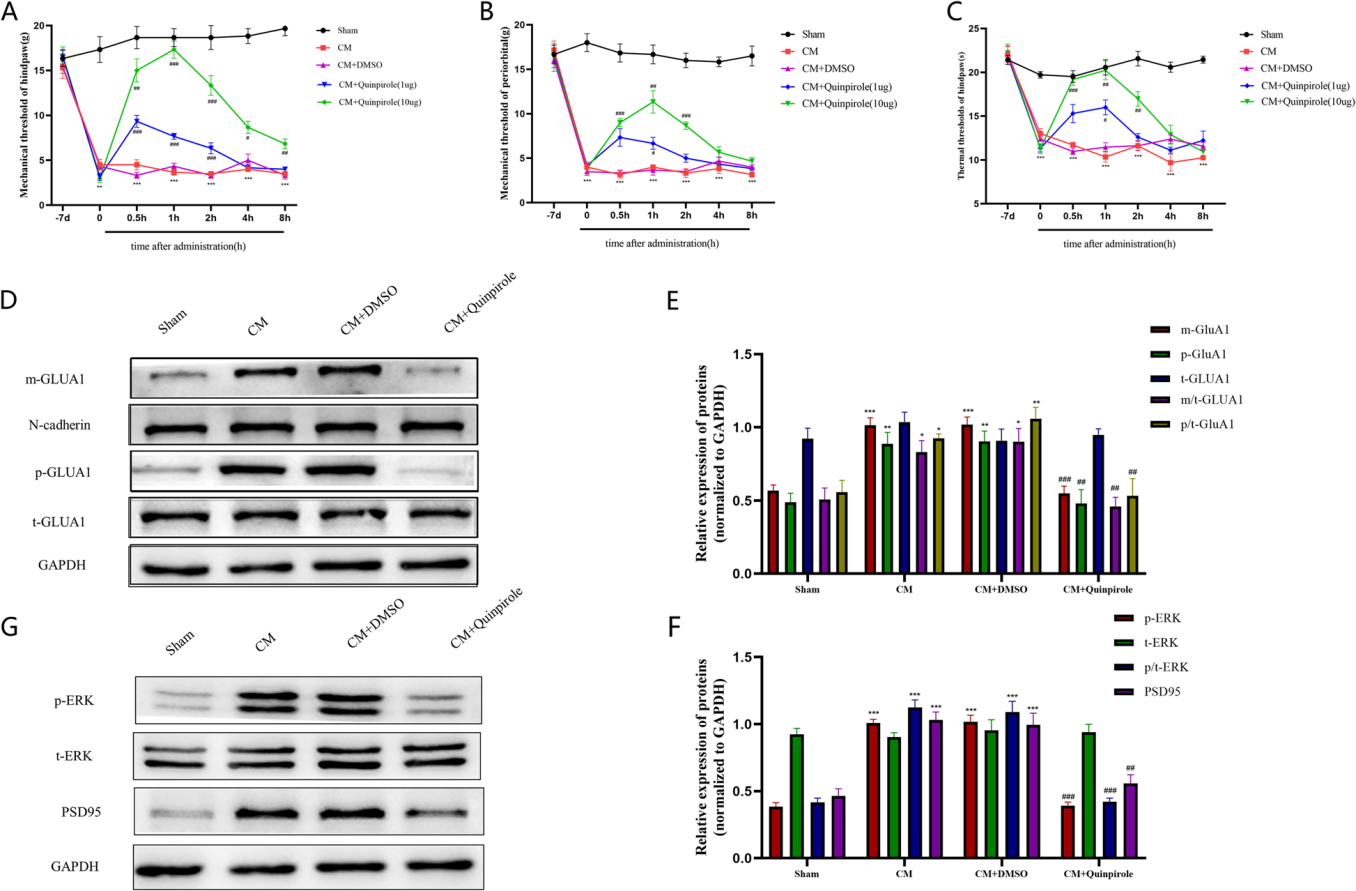
Effect of quinpirole on pain thresholds and GLUA1 trafficking in CM rats. **A B and C** Pain thresholds were increased after quinpirole (1 µg and 10 µg) administration in CM rats in a dose-dependent manner. Two-way ANOVA with the Bonferroni post hoc test. *n* = 6/group. **P < 0.01, ***P < 0.001 vs. the Sham group; #*P* < 0.05, ##*P* < 0.01, ###*P* < 0.001 vs. the CM + DMSO group. **D** and **E** Western blot showing enhanced GLUA1 trafficking and increased phosphorylation levels in the CM group, which were abolished by DRD2 agonist treatment; the total GLUA1 level was unchanged. p-GLUA1, m-GLUA1 and t-GLUA1 represent the phosphorylated, plasma membrane and total levels of GLUA1 respectively. p/t-GLUA1 represents the p-GLUA1/t-GLUA1 value; m/t-GLUA1 represents the (m-GLUA1/N-cadherin)/(t-GLUA1/GAPDH) value. **F** and **G** The increases in the protein levels of PSD95 and phosphorylated ERK in CM rats were dramatically inhibited by quinpirole, as shown by western blot analysis. One-way ANOVA with Dunnett’s post hoc test; *n* = 6/group. *P < 0.05, **P < 0.01, ***P < 0.001 vs. the Sham group; ##*P* < 0.01, ###*P* < 0.001 vs. the CM + DMSO group

To investigate the effect of DRD2 on pain behaviors in CM rats, CM rats were treated with different doses of the DRD2 agonist quinpirole (1 µg and 10 µg). As shown in Fig. 3A-C, the hind paw mechanical pain, thermal pain, and periorbital pain thresholds of CM rats were significantly elevated after quinpirole injection, indicating that DRD2 agonist treatment alleviated pain hypersensitivity in CM rats. In addition, the therapeutic effect of 10 µg quinpirole was greater than that of 1 µg quinpirole and was most obvious at 2 h postinjection and gradually diminished thereafter. And interestingly, the anti-hyperalgesic effect of quinpirole was effectively reversed by injecting sulpiride (an antagonist of DRD2) at 1 h after quinpirole injection (Additional File 1. S1A-C). Moreover, there was no effect of the administration of quinpirole on pain thresholds in Sham rats (Additional File 2. S2A-C).

Previous research has proven that the GLUA1 subunit is critical for the development of chronic migraine [43]; therefore, we next determined whether the pain-relieving effect of DRD2 is linked to GLUA1. First, we determined that DRD2 largely colocalized with GLUA1 in the TNC region in CM rats using double immunofluorescence staining (Fig. 2D). Subsequently, we used Western Blotting to examine the phosphorylation and plasma membrane expression levels of GLUA1. Consistent with the behavioral test results, the membrane insertion and phosphorylation of GLUA1 were enhanced in the CM group, and this enhancement was effectively inhibited by quinpirole treatment, while the total GLUA1 levels were unaffected (Fig. 3D-F).

We further examined the changes in the central sensitization-related protein ERK and the synapse-related protein PSD95 after quinpirole injection. Unsurprisingly, in the CM group, both the phosphorylation levels of ERK and the expression of PSD95 were greatly upregulated and statistically downregulated after quinpirole administration, while the level of total ERK was not significantly changed. (Fig. 3G, H).

### DRD2 antagonist treatment enhances central sensitization and GLUA1 trafficking in CM rats

**Fig. 4:**
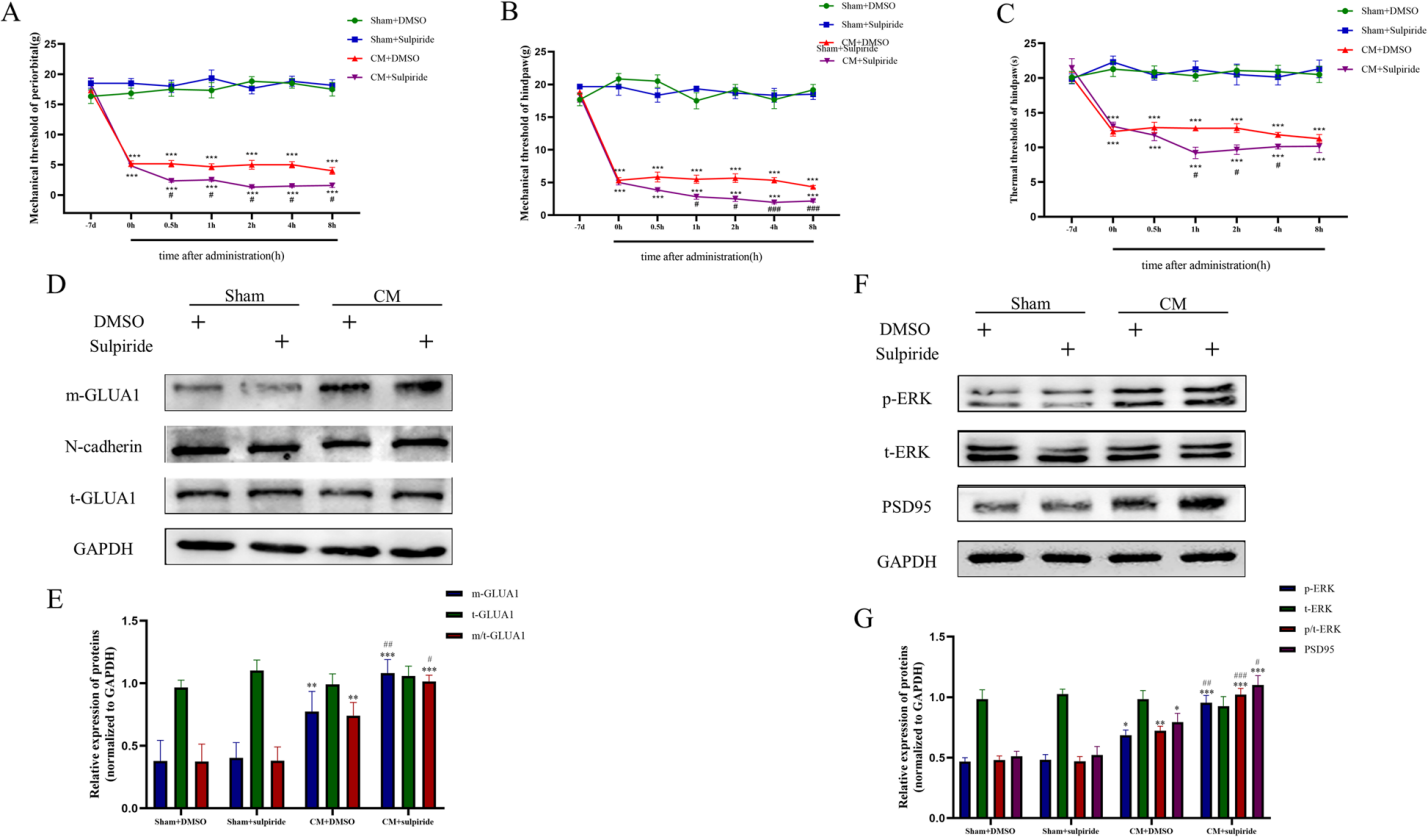
Effect of the DRD2 antagonist sulpiride on pain behaviors and GLUA1 trafficking. **A, B** and **C** The mechanical, thermal, and periorbital pain thresholds after sulpiride (30 µg) injection. Two-way ANOVA with the Bonferroni post hoc test; *n* = 6/group; ***P < 0.05 vs. the Sham group; #*P* < 0.05, ###*P* < 0.001 vs. the CM + DMSO group. **D**-**G**, Immunoblot analysis indicated that the protein levels of p-ERK and PSD95 and the plasma membrane level of GLUA1 were further elevated by sulpiride. One-way ANOVA with Dunnett’s post hoc test; *n* = 6/group; *P < 0.05, **P < 0.01, ***P < 0.001 vs. the Sham + DMSO group; #*P* < 0.05, ##*P* < 0.01, ###*P* < 0.001 vs. the CM + DMSO group

To further understand the role of DRD2 in CM rats, the effect of sulpiride (30 µg) on the pain thresholds in CM rats was tested. As shown in Fig. 4A-C, in contrast to the effects observed after quinpirole injection, the pain thresholds of CM rats decreased sharply after sulpiride injection but had no such effect in Sham rats, suggesting that sulpiride exacerbated pain hypersensitivity in CM rats. Moreover, GLUA1 trafficking was further enhanced after sulpiride injection in CM rats, as shown by western blotting (Fig. 4D, E). Next, the p-ERK and PSD95 levels were measured using western blotting to investigate the impact of sulpiride on central sensitization; as shown in Fig. 4F and G, the p-ERK and PSD95 levels in CM rats were significantly increased after sulpiride injection but not after 5% DMSO injection, indicating that inhibition of DRD2 can facilitate central sensitization in CM rats.

### NASPM treatment decreased allodynia and GLUA1-containing AMPAR trafficking in CM rats

**Fig. 5:**
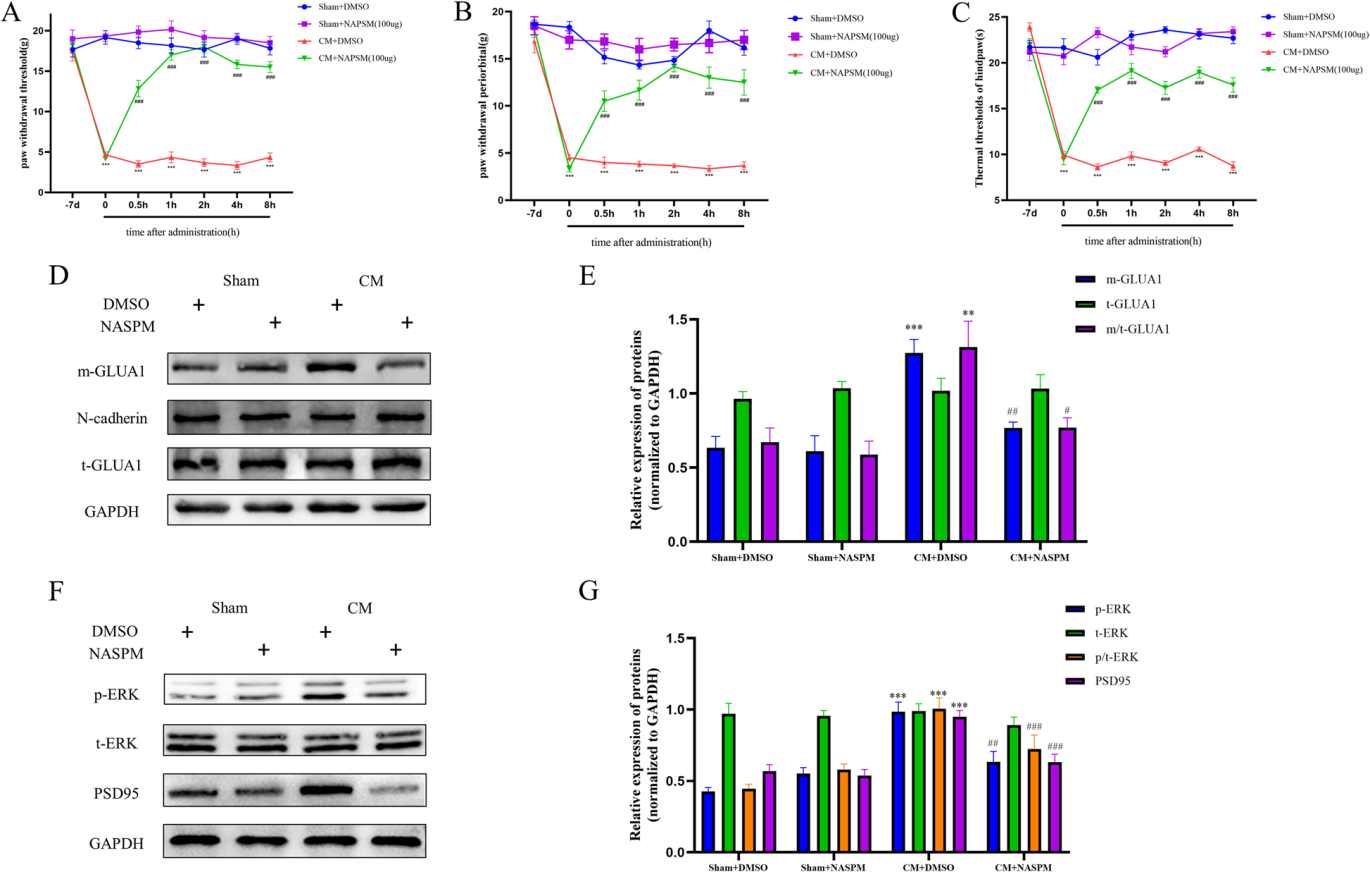
Blockade of GLUA1 attenuates central sensitization in CM rats. **A**-**C** Allodynia in CM rats was ameliorated by NASPM (100 µg). Two-way ANOVA with the Bonferroni post hoc test; *n* = 6/group; ***P < 0.001 vs. the Sham + DMSO group; ###*P* < 0.001 vs. the CM + DMSO group. **D**,** E** The enhanced GLUA1 trafficking in CM rats was reversed by NASPM as shown by western blot analysis. **F**, **G** p-ERK and PSD95 levels were decreased after NASPM administration. One-way ANOVA with Dunnett’s post hoc test; *n* = 6/group; **P < 0.01, ***P < 0.001 vs. the Sham + DMSO group; #*P* < 0.05, ##*P* < 0.01, ###*P* < 0.001 vs. the CM + DMSO group

To further determine the modulatory effect of GLUA1-containing AMPARs on chronic migraine, NASPM (100 µg) was applied to specifically block GLUA1-containing AMPARs [44]. In CM rats, administration of NASPM alleviated the established mechanical, periorbital, and thermal pain hypersensitivity, but no such effect on pain thresholds was observed in the Sham group (Fig. 5A-C). Interestingly, NASPM specifically inhibited the aggregation of GluA1-containing AMPARs on the postsynaptic surface without affecting total protein expression in the TNC in CM rats (Fig. 5D, E). More importantly, administration of NAPSM decreased the PSD95 protein level and reduced ERK phosphorylation in CM rats. Consistent with the behavioral test results, regulation of PSD95 and ERK by NAPSM was not observed in the Sham group (Fig. 5F, G).

### GLUA1 trafficking in CM rats requires the PI3K signaling pathway

**Fig. 6:**
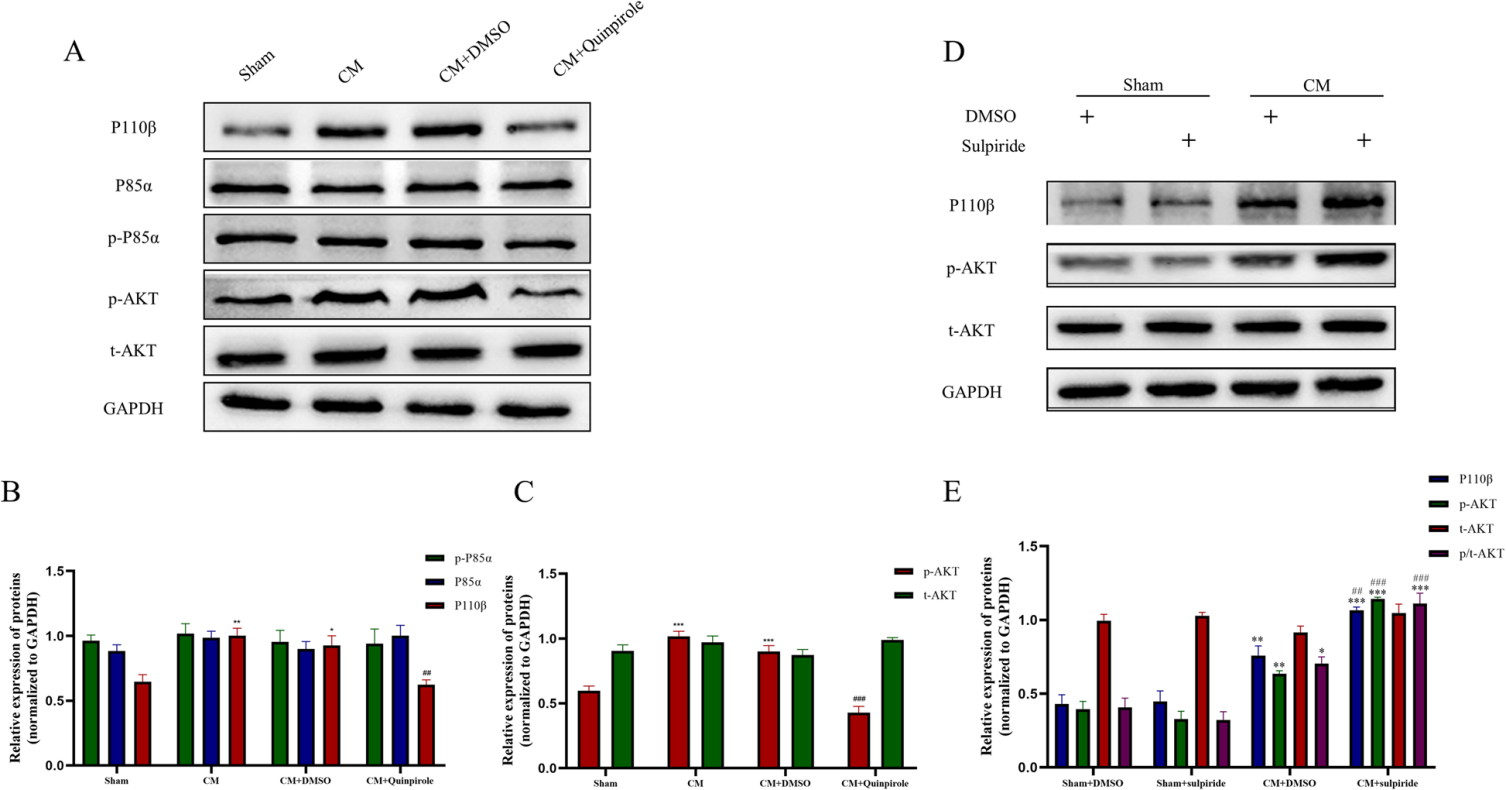
Effects of the DRD2 agonist quinpirole and DRD2 antagonist sulpiride on the PI3K pathway in CM rats. **A**, **B** and **C**, The levels of AKT phosphorylation and PI3K P110β subunit were significantly increased in the CM group, and this activation was reversed by DRD2 agonism. No marked changes in the total and phosphorylated P85α subunit protein levels and total AKT protein levels among the groups were observed. One-way ANOVA with Dunnett’s post hoc test; *n* = 6/group; *P < 0.05, **P < 0.01, ***P < 0.001 vs. the Sham group; ##*P* < 0.01, ###*P* < 0.001 vs. the CM + DMSO group. **D** and **E**, western blotting analysis showed that sulpiride dramatically increased PI3K P110β submit and phosphorylated AKT protein levels in CM rats. One-way ANOVA with Dunnett’s post hoc test; *n* = 6/group; *P < 0.05, **P < 0.01, ***P < 0.001 vs. the Sham + DMSO group; ##*P* < 0.01, ###*P* < 0.001 vs. the CM + DMSO group

We first explored PI3K/AKT pathway activation and the effect of quinpirole on the PI3K/AKT pathway in CM rats. Western Blotting analysis showed that the total abundance and phosphorylation of the PI3K P85α-subunit were not significantly different among the groups, while the phosphorylation levels of AKT and the PI3K P110β-subunit were markedly increased in the CM group and reduced by quinpirole treatment (Fig. 6A-C). Additional results revealed that the Sham rats after quinpirole injection showed no significant changes in the PI3K pathway, which indicate that quinpirole had no effect on the PI3K signaling in the absence of IS stimulation (Additional File 2. S2D-G). Interestingly, the PI3K P110β and p-AKT levels were significantly elevated in the CM + sulpiride group compared with the CM + DMSO group, while these increases were not observed in the Sham group (Fig. 6D, E). These observations demonstrate that the PI3K/AKT pathway, which can be regulated by DRD2, is essential in the development of CM.

**Fig. 7:**
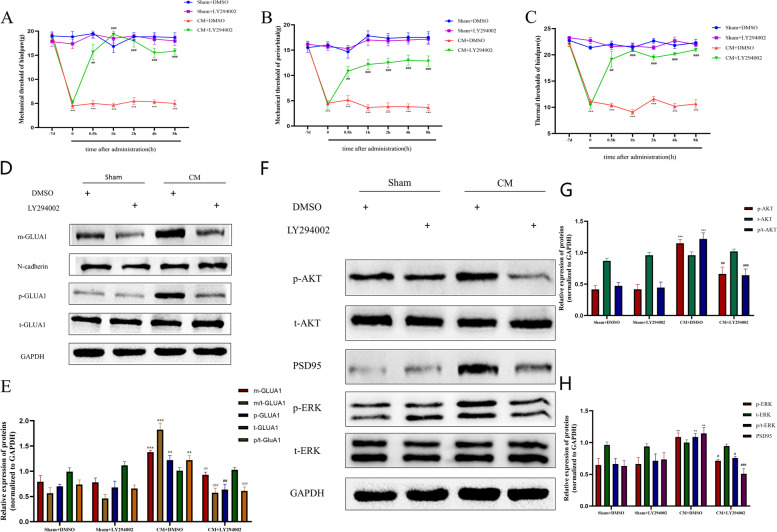
Effects of the PI3K pathway inhibitor LY294002 on GLUA1 trafficking and central sensitization in CM rats. **A**, **B**, and **C** Pharmacological inhibition of the PI3K pathway significantly alleviated pain hypersensitivity in CM rats. Two-way ANOVA with the Bonferroni post hoc test; *n* = 6/group; **P < 0.01, ***P < 0.001 vs. the Sham + DMSO group; ##*P* < 0.01, ###*P* < 0.001 vs. the CM + DMSO group. **D** and **E** PI3K inhibition inhibited GLUA1 trafficking and reduced the p-GLUA1 level, which were increased in CM rats. C D and E Protein levels of PSD95, phosphorylated AKT and phosphorylated ERK were decreased after LY294002 injection in CM rats compared to CM + DMSO rats. There were no significant changes in the total ERK and AKT protein levels. One-way ANOVA with Dunnett’s post hoc test; *n* = 6/group; **P < 0.01, ***P < 0.001 vs. the Sham + DMSO group; #*P* < 0.05, ##*P* < 0.01, ###*P* < 0.001 vs. the CM + DMSO group

Subsequently, the effect of the PI3K inhibitor LY294002 (10 µg) on pain thresholds was analyzed. As shown in Fig. 7A-C, LY294002 significantly alleviated pain hypersensitivity in CM rats compared to control rats, and this effect appeared 30 min after administration and was sustained for at least 8 h. As expected, there was no significant effect of the drug vehicle on pain thresholds and no significant effect of LY294002 on pain thresholds in the Sham group.

We then examined whether PI3K/AKT signaling functions in membrane recruitment of GLUA1 in CM rats. Western blot analysis showed that LY294002 dramatically decreased the phosphorylation and trafficking of GLUA1, whereas this phenomenon was not observed in the sham group (Fig. 7D, E). Then we investigated the effect of LY294002 on ERK and PSD95 and found that LY294002 significantly reduced the p-ERK and PSD95 levels (Fig. 7F-G).

### DRD2 agonist treatment attenuates central sensitization and reduces membrane expression of GLUA1 via the PI3K pathway

**Fig. 8:**
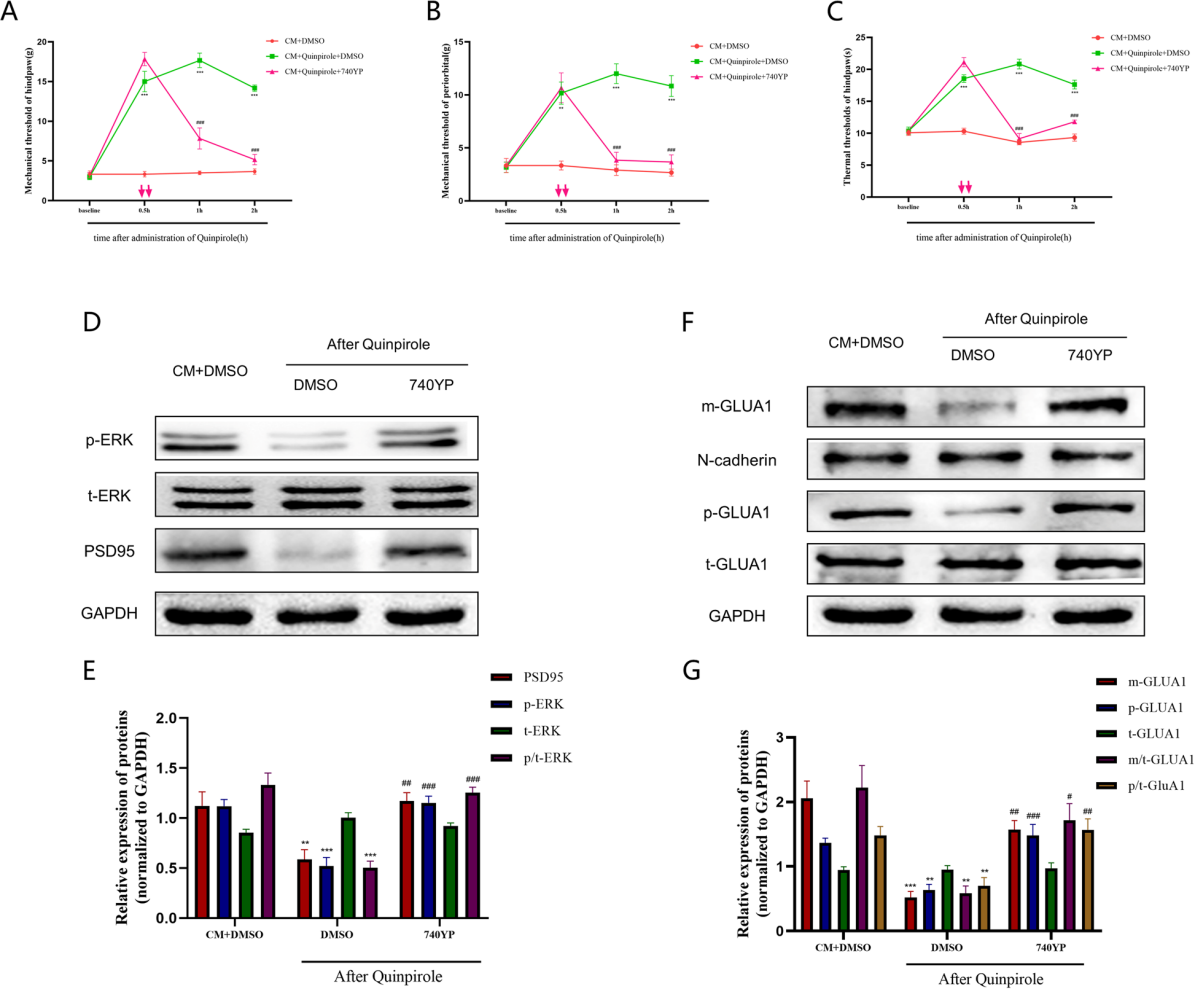
DRD2 agonist treatment attenuates central sensitization via the PI3K signaling pathway. **A**, **B** and **C** The PI3K activator 740YP reversed the anti-injury effects of the DRD2 agonist quinpirole and reduced pain thresholds in CM rats. Two-way ANOVA with the Bonferroni post hoc test; *n* = 6/group; **P < 0.01, ***P < 0.001 vs. the Sham + DMSO group; ###*P* < 0.001 vs. the CM + DMSO group. **D** and **E** 740YP reversed the DRD2-mediated reductions in the protein expression of PSD95 and phosphorylation of ERK. **F** and **G** 740YP abolished the effects of the DRD2 agonist on reducing GLUA1 membrane trafficking and p-GLUA1 levels. One-way ANOVA with Dunnett’s post hoc test; *n* = 6/group; **P < 0.01, ***P < 0.001 vs. the Sham + DMSO group; #*P* < 0.05, ##*P* < 0.01, ###*P* < 0.001 vs. the CM + DMSO group

To determine whether DRD2 attenuates central sensitization via a mechanism dependent on the PI3K/AKT pathway, we administered the PI3K-specific agonist 740YP to CM rats 1 h after quinpirole injection and subsequently assessed the pain behavior of the rats. Strikingly, the antinociceptive effect of quinpirole was substantially counteracted by 740YP (Fig. 8A-C), and consistent with the results of the pain behavioral tests, GLUA1 phosphorylation and membrane expression in CM rats were significantly reduced after quinpirole injection. Interestingly, these effects were effectively reversed by 740YP, and the total amount of GLUA1 remained unaffected (Fig. 8D, E). These results imply that DRD2 agonist treatment reduces pain behaviors and GLUA1 membrane trafficking in CM rats via a mechanism largely dependent on the PI3K signaling. In addition, the changes in the phosphorylation of ERK and the expression of PSD95 were significantly reversed after 740YP injection (Fig. 8F, G).

**Fig. 9:**
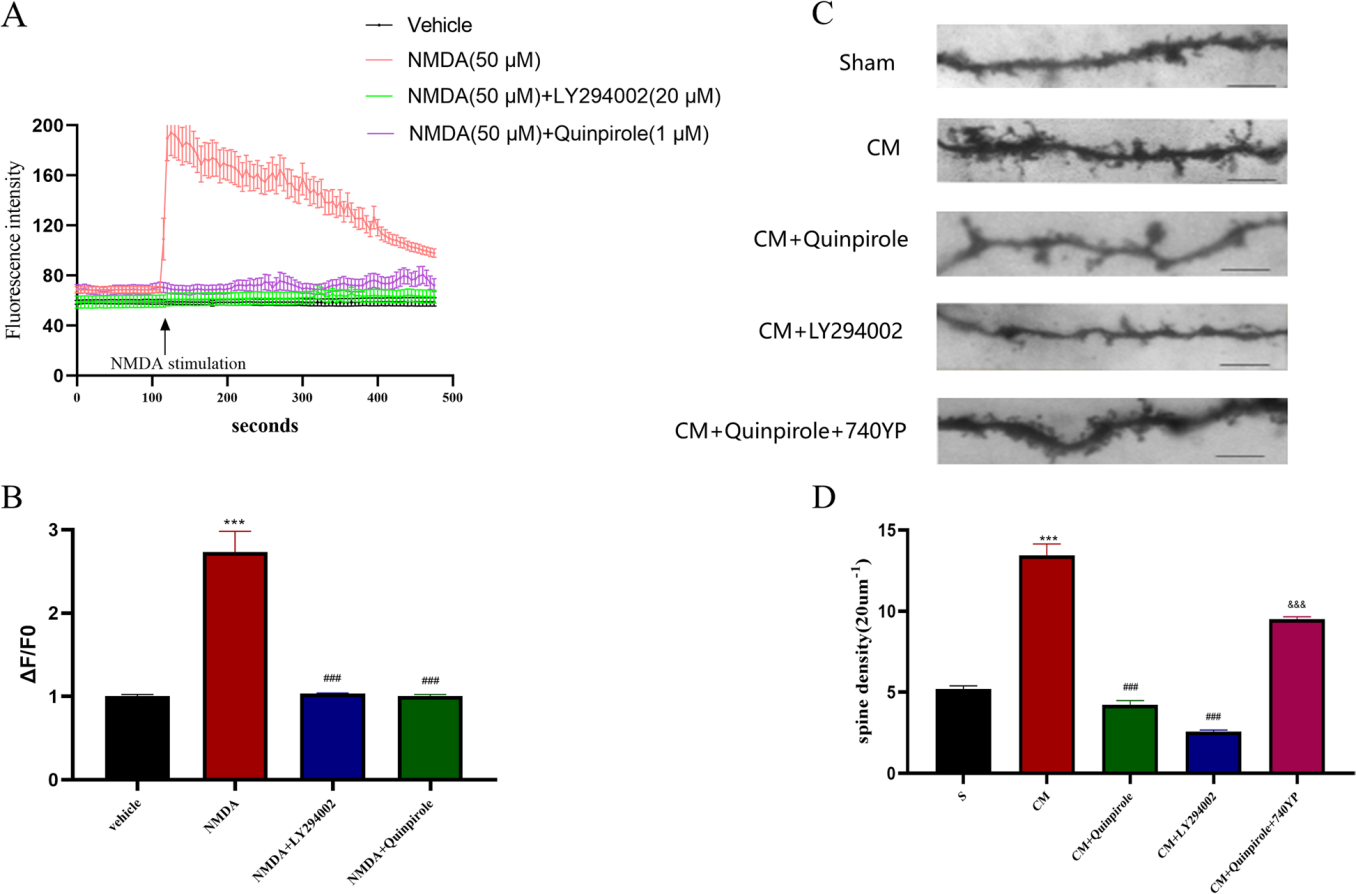
Effects of DRD2 and the PI3K pathway on the dendritic spine density and calcium concentration. **A** and **B** Representative traces, and quantitative analysis of the effects of quinpirole (1 μM) and LY294002(20 μM) on the NMDA (50 μM)-induced increase in Ca2 + in TNC neurons cultured in vitro. Neurons were pre-treated with quinpirole for 12 h and LY294002 for 1 h at 37℃ and 5% CO2. One-way ANOVA with Dunnett’s post hoc test; *n* = 20 cells/group; ***P < 0.001 vs. the Vehicle group; ###*P* < 0.001 vs. the NMDA group. **C** and **D** Representative photomicrographs of spine morphology in the TNC in CM rats are shown (scale bar = 5 mm); The dendritic spine density was sharply increased in the CM group compared with the Sham group; this increase was abolished by both the DRD2 agonist quinpirole and the PI3K inhibitor LY294002, and the therapeutic effect of quinpirole was reversed by the PI3K activator 740YP. One-way ANOVA with Dunnett’s post hoc test; *n* = 5/group; ***P < 0.001 vs. the Sham group; ###*P* < 0.001 vs. the CM group, &&&*P* < 0.001 vs CM + LY294002 group

Considering that the process of central sensitization is accompanied by persistent calcium influx, we then explored the impact of DRD2 and the PI3K/AKT pathway on the intraneuronal calcium concentration. In agreement with previous studies, we established a model of neuronal excitation by applying NMDA (50 µM) to primary neurons cultured in vitro. We found that NMDA significantly stimulated neuronal calcium influx, which was effectively inhibited by both quinpirole and LY294002 (Fig. 9A, B). The above results suggest that DRD2 contributes greatly to attenuating central sensitization in CM rats and that this effect is at least partially dependent on PI3K signaling.

### DRD2 and the PI3K pathway regulate the dendritic spine density and synaptic ultrastructure at the TNC in CM rats

Dendritic spines are the main postsynaptic sites for excitatory synaptic inputs, which are important factors in synaptic plasticity and function. In response to noxious stimuli, the density and complexity of dendritic spines are altered accordingly, and these alterations are the basis of synaptic plasticity and central sensitization. Therefore, Golgi-Cox staining was used to assess the influence of DRD2 and the PI3K pathway on dendritic spine morphology in CM rats. According to the data, the density of dendritic spines was massively increased in CM rats (Fig. 9C, D). Not surprisingly, both quinpirole and LY294002 significantly suppressed the increase in the dendritic spine density in CM rats. Interestingly, the inhibitory effect of quinpirole was once again counteracted by the PI3K agonist 740YP (Fig. 9).

**Fig. 10:**
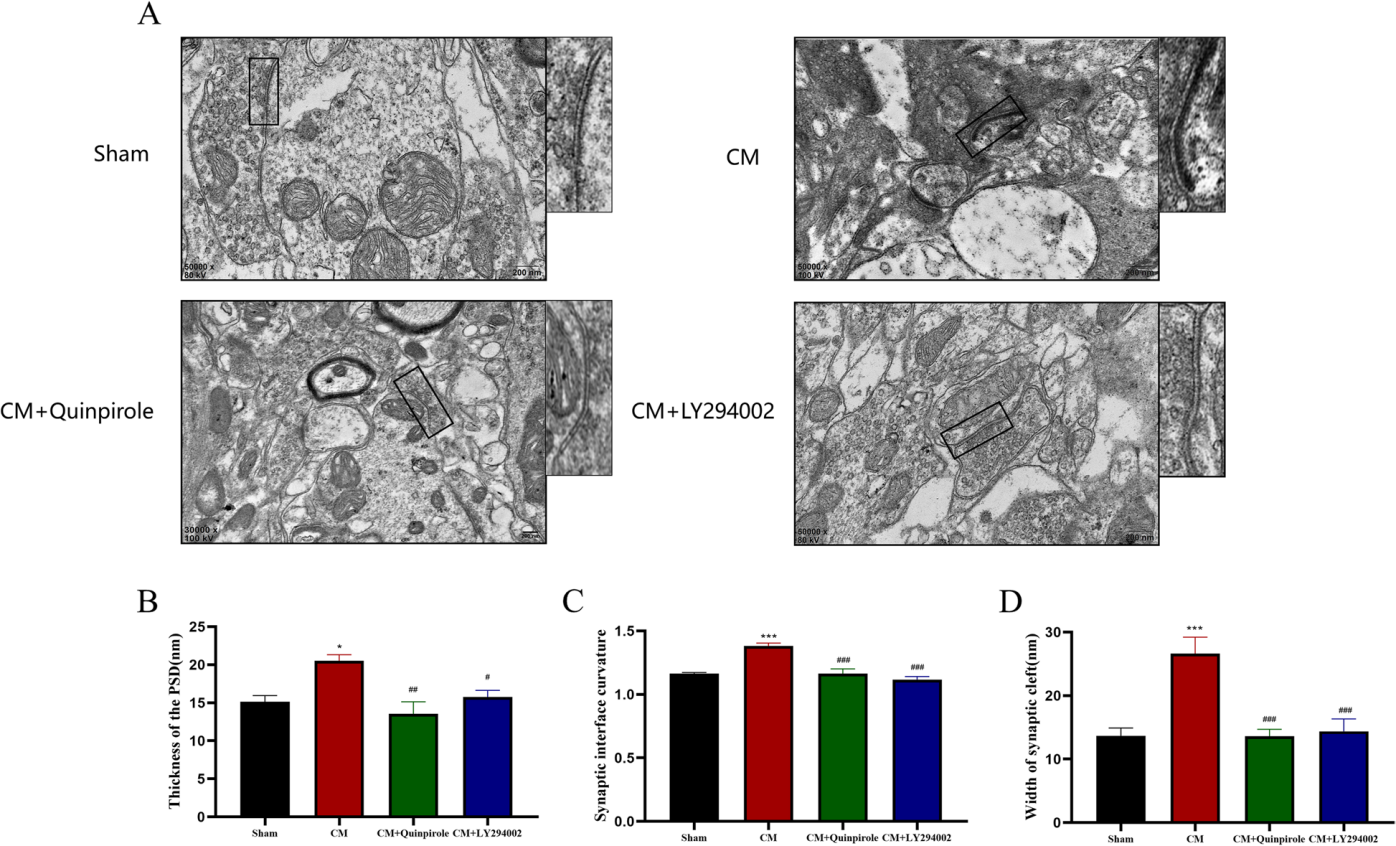
Regulation of the synaptic ultrastructure of neurons in the TNC in CM rats by DRD2 and the PI3K pathway. **A** representative electroscopic image of the synaptic ultrastructure in each group (Scale bar = 200 nm). **B**, **C**, and **D** Rats in the CM group had a thicker PSD(B), a higher synaptic interface curvature(C), and a wider synaptic cleft(D) than rats in the Sham group, and these changes were counteracted by both quinpirole and LY294002. One-way ANOVA with Dunnett’s post hoc test; *n* = 5/group; *P < 0.05, ***P < 0.001 vs. the Sham group; #*P* < 0.05, ##*P* < 0.01, ###*P* < 0.001 vs. the CM group

The development of central sensitization is accompanied not only by changes in dendritic spines but also by changes in the synaptic ultrastructure, including the synaptic cleft, the synaptic interface, and the postsynaptic dense zone (PSD) [40]. Here, we used transmission electron microscopy to observe modifications to the synaptic ultrastructure in CM rats. Similar to the above results, CM rats showed significant narrowing of the synaptic cleft, thickening of the postsynaptic dense zone, and increased curvature of the synaptic interface, and these changes were effectively reversed by quinpirole and LY294002 (Fig. 10A-D).

### DRD2 mediates the activation of the PI3K pathway probably via Src family kinases

**Fig. 11:**
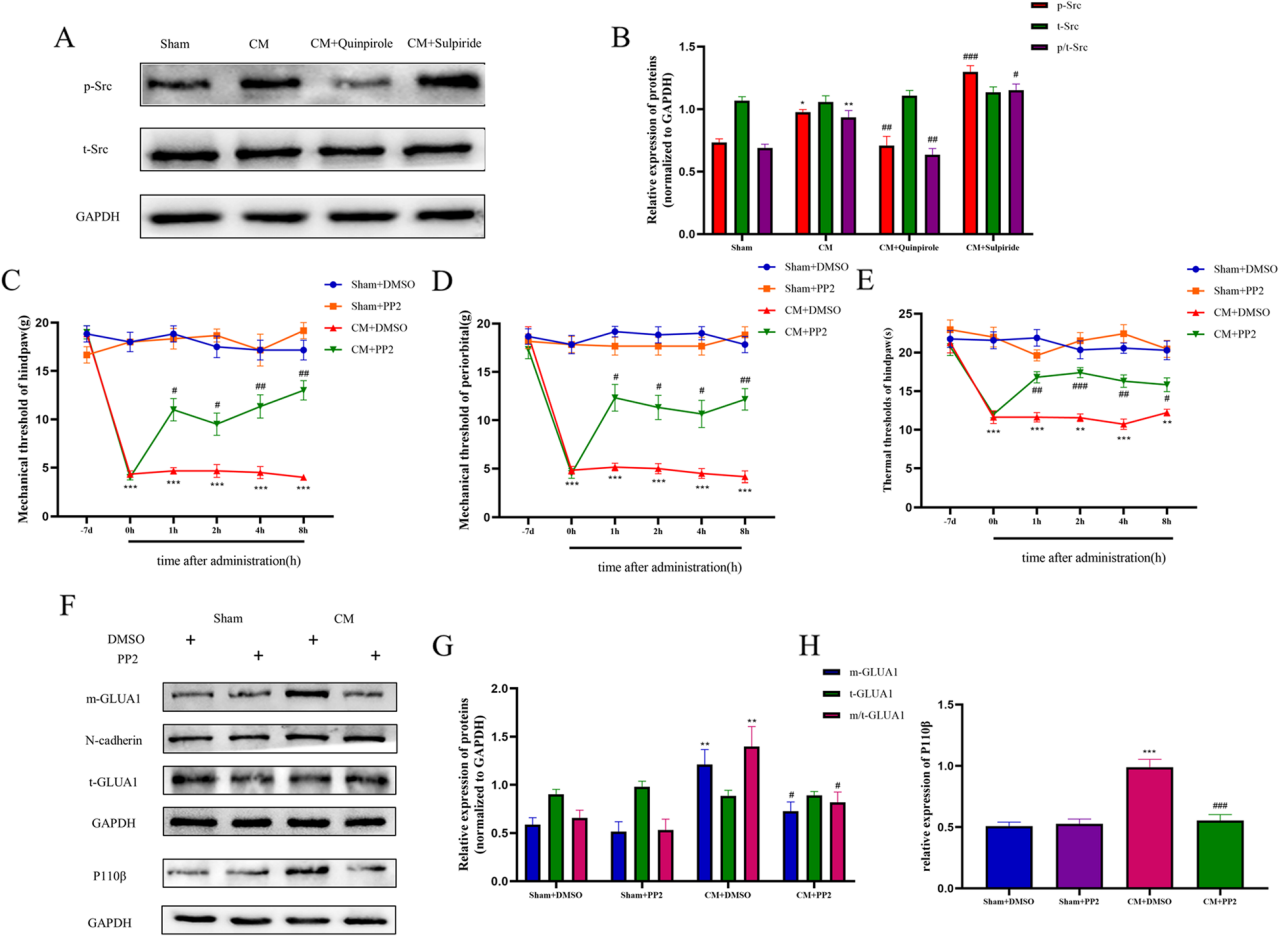
Src family kinases may mediate the regulation of the PI3K signaling pathway by DRD2. **A** and **B** The p-SFK level was increased in CM rats and was decreased by quinpirole and further upgraded by sulpiride. p/t-SFK indicates the p-SFK/t-SFK value. One-way ANOVA with Dunnett’s post hoc test; n = 6/group; *P < 0.05, **P < 0.01 vs. the Sham group; #*P* < 0.05, ##*P* < 0.01, ###*P* < 0.001 vs. the CM group. **C**-**E** PP2(a Src family kinase inhibitor) relieved pain associated with chronic migraine. *n* = 6/group; Two-way ANOVA with the Bonferroni post hoc test. **F**, **G** Western blot analysis revealed that PP2 inhibited PI3K signaling activation and GLUA1 trafficking. One-way ANOVA with Dunnett’s post hoc test; *n* = 6/group; **P < 0.01, ***P < 0.001 vs. the Sham + DMSO group; #*P* < 0.05, ###*P* < 0.001 vs. the CM + DMSO group

To fully explore the possible mechanism by which DRD2 regulates the PI3K pathway, we focused on Src family kinases. As shown in Fig. 11A and B, the phosphorylation levels of Src kinase were significantly increased in CM rats and were regulated by quinpirole and sulpiride. These results indicate that the Src family kinases can be regulated by DRD2 and may play an important role in CM rats. Next, we studied the effect of PP2 (a Src family inhibitor) on pain hypersensitivity in CM rats. Consistent with previous studies, pain hypersensitivity in CM rats was significantly reversed after PP2 injection, without any effect on the pain thresholds in Sham rats (Fig. 11C-E). In addition, western blotting showed that PP2 treatment decreased GLUA1 trafficking (Fig. 11F, G). Additionally, further results suggested that the inhibition of Src also downregulated the expression of PI3K P110β (Fig. 11F, H). Taken together, these results indicate that the regulation of the PI3K pathway by DRD2 may be mediated via a mechanism involving Src family kinases.

## Discussion

The anti-injury effects of DRD2 and its probable mechanisms of action in rats with IS-induced chronic migraine were examined in this paper. We demonstrated that (1) the periorbital, hind paw mechanical, and thermal pain thresholds were markedly reduced in rats after 7 consecutive days of IS titration, accompanied by a decrease in DRD2 expression and an increase in the membrane insertion of GLUA1-containing AMPARs in the TNC; (2) both the DRD2 agonist quinpirole and the PI3K inhibitor LY293002 increased the pain thresholds, inhibited the membrane translocation of GLUA1-containing AMPARs and reversed the alterations in the dendritic spine density as well as the synaptic ultrastructure, thereby attenuating central sensitization in CM rats; (3)the DRD2 inhibitor sulpiride exacerbated central sensitization and GLUA1 trafficking in CM rats; (4) Blockade of GLUA1 by NASPM reduced GLUA1 trafficking and attenuates central sensitization in CM rats; (5) agonism of DRD2 and inhibition of the PI3K pathway inhibited NMDA-induced calcium influx in cultured primary neurons in vitro; (6) DRD2 agonist alleviated pain behaviors, inhibited the membrane transport of GLUA1-containing AMPARs and reduced central sensitization in CM rats, probably through the PI3K pathway; and (7) DRD2 regulated PI3K signaling possibly through a mechanism involving Src family kinases. These results imply that DRD2 may play a substantial role in the pathophysiological process of chronic migraine and therefore could be a reasonable pharmaceutical candidate for the treatment of chronic migraine.

The rat CM model was established by infusing IS and stimulating the trigeminal vascular system using an internationally approved and reliable surgical cannula [35, 36]. Estrogen is strongly associated with migraine attacks, as has been reported previously [45, 46]. Estrogen has a complex relationship with the function of the dopamine system in the brain; females have been reported to have a more active dopamine system than males, which may have unanticipated implications for this study [46, 47]. In addition, rodents with migraine show great differences in nociceptive processing [48]. Therefore, in the present study, male rats were eventually chosen to control for possible bias introduced by the presence of estrogen.

As a part of the descending nociceptive inhibitory system, the role of the dopamine system in chronic migraine has gradually garnered research focus [35, 49]. The involvement of dopamine receptors in chronic migraine has been widely reported, and bioactive ingredients derived from traditional Chinese medicine have been reported to alleviate migraine by modulating dopamine receptors [49–51]. However, the changes in dopamine receptor expression in CM and how they function are still not fully explored. In the present research, we reported that DRD2 was downregulated in CM rats and that the application of a DRD2 agonist alleviated pain behaviors and central sensitization in CM rats, while the administration of a DRD2 antagonist had the opposite effect. These results are certainly encouraging; the underlying mechanisms, however, have rarely been discussed. In addition, this study focused on the mechanism by which DRD2 in the TNC acts to alleviate chronic migraine. The alterations and functions of the dopamine system in other regions of the brain, particularly those of the descending pain inhibitory system in CM rats will be addressed in future research.

Postsynaptic membrane recruitment of GLUA1-containing AMPARs but not GLUA2/3-containing AMPARs is a crucial step in the transmission of nociceptive messages and central sensitization [8, 52]. It has been previously reported that GLUA1-containing AMPARs in the TNC contribute greatly to chronic migraine [9, 43]. In this study, we confirmed that inhibition of GLUA1 trafficking attenuates central sensitization and pain hyperesthesia in CM rats. We also clarified that DRD2 acts as an important upstream factor of GLUA1-containing AMPARs and is an important factor regulating central sensitization in CM rats. Consistent with earlier results, we found that the increased recruitment and phosphorylation level of GLUA1-containing AMPARs in the TNC in CM rats was reversed by the administration of quinpirole (a DRD2-specific agonist) and enhanced by sulpiride (a DRD2 antagonist). However, the potential links between DRD2 and GLUA1-containing AMPARs in CM rats remain uncertain.

Phosphatidylinositol 3-kinase (PI3K) is commonly expressed in various brain areas and is essential in the onset and maintenance of chronic pain [27, 53, 54]. In addition, the PI3K/AKT signaling pathway has been reported to modulate the synaptic plasticity of neurons and the phosphorylation of GLUA1-containing AMPARs and their distribution at synapses [25, 26]. Our study revealed that the expression of the PI3K P110β subunit was increased in CM rats. Inhibition of the PI3K/AKT pathway significantly alleviated pain behaviors and inhibited the phosphorylation and membrane surface expression of GLUA1-containing AMPAR in CM rats. Here, we hypothesized that DRD2 attenuates central sensitization in CM in association with the PI3K pathway, and this hypothesis was confirmed by our finding that the aberrant activation of the PI3K/AKT pathway was inhibited by the DRD2 agonist quinpirole but further augmented by the DRD2 antagonist sulpiride. In addition, the PI3K agonist 740YP abolished the anti-injury effect of the DRD2 agonist quinpirole. Moreover, the effects of DRD2 on inhibiting GLUA1-containing AMPAR translocation to the synaptic membrane and attenuating central sensitization were disrupted by 740YP. Overall, our findings indicate that there is a functional link between DRD2 and the PI3K pathway and that the PI3K pathway plays a critical hub role in the inhibitory effects of DRD2 on GLUA1-containing AMPAR postsynaptic recruitment, central sensitization, and nociceptive transmission. Furthermore, our results indicate that Src family kinases are probably the mediators linking DRD2 and the PI3K signaling pathway.

In general, the development of central sensitization is usually accompanied by enhanced neuronal transmission and structural plasticity, including the plasticity of dendritic spines and enhanced synaptic connections [55–57]. Therefore, in this study, to verify the impact of DRD2 and the PI3K pathway on central sensitization, in addition to using western blotting to detect ERK and PSD95 expression, we used Golgi-Cox staining and transmission electron microscopy to visualize dendritic spine complexity and the synaptic ultrastructure, and Fluo-4-AM was used to monitor the calcium concentration. The results provided strong evidence for the influential roles of DRD2 and PI3K signaling in regulating neuronal excitability and central sensitization [58–60], in cultured primary neurons in vitro. On the other hand, excitatory postsynaptic currents mediated by glutamate receptors drive central sensitization and the progression of chronic pain [5, 56, 57]. However, the effects of DRD2 and the PI3K pathway on electrophysiological activity in the TNC in CM rats have not been explored, which is a limitation of this study and will be our focus in future studies. In addition, we used NMDA instead of AMPA to establish the model of neuronal excitation [32] taking into account the neurotoxicity mediated by AMPA [61] and the observation that NMDA can also induce the increased expression of GLUA1-containing AMPARs on the membrane surface of neurons, which is pertinent to the purpose of this study [18, 62].  

## Conclusion

In summary, agonism of D2DR in the TNC regulates GluA1-containing AMPAR trafficking, alleviates pain behaviors and attenuates central sensitization in CM model rats, thereby relieving IS-induced chronic migraine. Further results suggest that this effect is mediated, at least in part, through the PI3K pathway. These results shed light on the pathophysiology of chronic migraine and emphasize that D2DR could be a possible therapeutic candidate for the relief of chronic migraine.

## Supplementary Information

**Additional file 1:** **Figure S1.** Sulpiride abolished the anti-injury effect of quinpirole treatment. A, B,and C, The DRD2 antagonist sulpiride (30 μg) reversed theanti-injury effects of the DRD2 agonist quinpirole (sulpiride was injected at 1h after quinpirole treatment, 5% DMSO as the vehicle). Two-way ANOVA with theBonferroni post hoc test; *n* = 6/group;*P<0.05, **P<0.01, ***P <0.001 vs. the CM+DMSO group; ##*P*<0.01,###*P*<0.001 vs. the CM+quinpirole+DMSOgroup.

**Additional file 2:** **Figure S2.** Effect of quinpirole on Sham rats. A, B, and C, quinpirole(10 μg) had no effect on pain thresholds in the Sham rats. *n* = 6/group; Two-way ANOVA with the Bonferroni post hoc test. D, E, F, and G, western blottinganalysis revealed that GLUA1 trafficking, protein levels of P110β and p-AKT,and p-ERK levels were not regulated by quinpirole treatment in Sham rats. *n* = 6/group; One-way ANOVA with Dunnett’s post hoc test.

## Data Availability

The datasets used and analyzed during the current study are available from the corresponding author on reasonable request.

## Abbreviations

CM: Chronic migraine
CNS: Central nervous system
DRD2: Dopamine D2 receptor
SFK: Src family kinases
AMPAR: α-Amino-3-hydroxy-5-methyl-4-isoxazolepropionic acid receptor
NMDAR: N-methyl-D-aspartate receptor
GPCRs: G protein-coupled receptors
PWL: Paw twitch latency
TNC: Trigeminal nucleus caudalis
PI3K: Phosphoinositide-3-kinase
TEM: Transmission electron microscope
LTP: Long-term potentiation
GAPDH: Glyceraldehyde-3-phosphate dehydrogenase
qRT‒PCR: Quantitative real-time polymerase chain reaction
WB: Western blot
PVDF: Polyvinylidene difluoride
TBST: Tris-buffered saline containing Tween 20
PBS: Phosphate-buffered saline
