# Supplementary figures and images for: Dopamine receptor D2 regulates GLUA1-containing AMPA receptor trafficking and central sensitization through the PI3K signaling pathway in a male rat model of chronic migraine
Source: J Headache Pain. 2022 Aug 10;23(1):98. doi: 10.1186/s10194-022-01469-x (PMC9364568; doi:10.1186/s10194-022-01469-x)

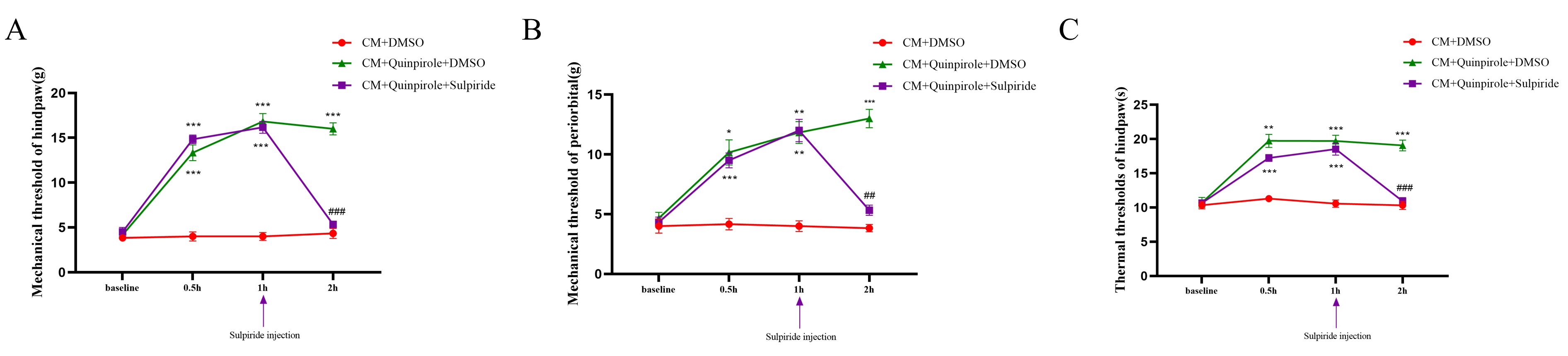

Supplement: Supplementary file 1 — Additional file 1: Figure S1. Sulpiride abolished the anti-injury effect of quinpirole treatment. A, B,and C, The DRD2 antagonist sulpiride (30 μg) reversed theanti-injury effects of the DRD2 agonist quinpirole (sulpiride was injected at 1h after quinpirole treatment, 5% DMSO as the vehicle). Two-way ANOVA with theBonferroni post hoc test; n = 6/group;*P<0.05, **P<0.01, ***P <0.001 vs. the CM+DMSO group; ##P<0.01,###P<0.001 vs. the CM+quinpirole+DMSOgroup. [file 10194_2022_1469_MOESM1_ESM.tif]

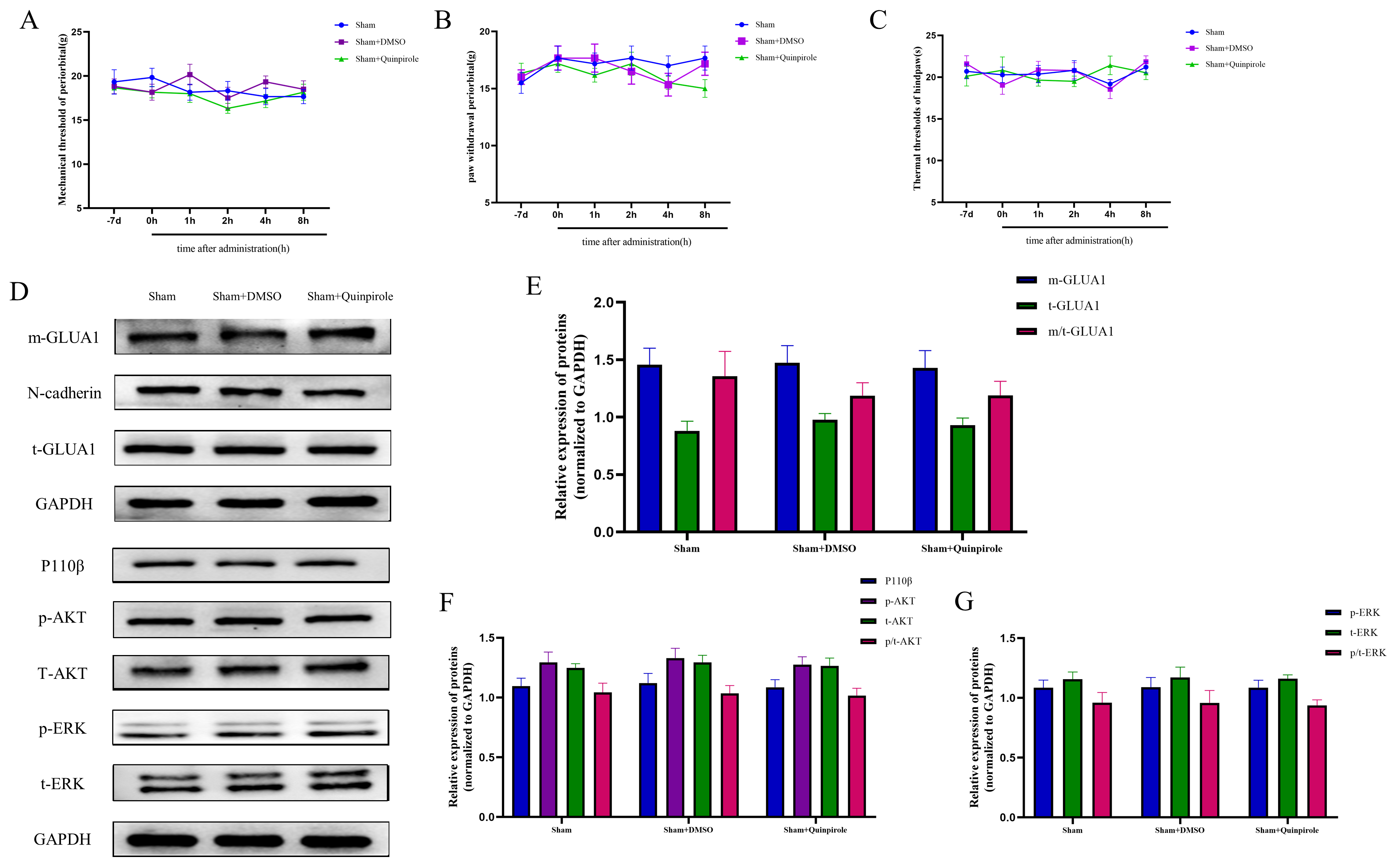

Supplement: Supplementary file 2 — Additional file 2: Figure S2. Effect of quinpirole on Sham rats. A, B, and C, quinpirole(10 μg) had no effect on pain thresholds in the Sham rats. n = 6/group; Two-way ANOVA with the Bonferroni post hoc test. D, E, F, and G, western blottinganalysis revealed that GLUA1 trafficking, protein levels of P110β and p-AKT,and p-ERK levels were not regulated by quinpirole treatment in Sham rats. n = 6/group; One-way ANOVA with Dunnett’s post hoc test. [file 10194_2022_1469_MOESM2_ESM.tif]
